# Neointimal coverage after second generation drug-eluting stent implantation has a relationship with pre-existing atherosclerotic lesion characteristics

**DOI:** 10.1097/MD.0000000000017097

**Published:** 2019-09-13

**Authors:** Yohta Nomoto, Masashi Nakagawa, Nobuyuki Shirai, Keiko Kajio, Kazuki Mizutani, Takanori Yamazaki, Kenichi Sugioka, Kimio Kamimori, Makiko Ueda, Yasuhiro Izumiya, Minoru Yoshiyama

**Affiliations:** a Department of Cardiovascular Medicine, Osaka City University Graduate School of Medicine; b Department of Cardiology, Osaka City General Hospital; c Morinomiya University of Medical Science, Osaka, Japan.

**Keywords:** drug-eluting stent, neointimal thickness, optical coherence tomography, plaque characteristic

## Abstract

The relationship between preexisting atherosclerotic lesion characteristics and neointimal thickness after second-generation drug-eluting stent (DES) placement is still unknown. Thus, we evaluated that relationship using optical coherence tomography (OCT).

A single-center, retrospective, observational study was conducted. Patients with stable angina or asymptomatic myocardial ischemia who received percutaneous coronary intervention for a de novo lesion using a second-generation DES under frequency domain OCT guidance and underwent follow-up coronary angiography (CAG) and OCT between December 2010 and December 2015 were included. The relationship between the neointimal thickness on the stent strut and the plaque characteristics was retrospectively evaluated using OCT immediately after stent implantation and at the time of follow-up CAG.

We analyzed 3459 struts from 20 stents in 15 patients. The mean follow-up period was 264 days. In the follow-up study, no angiographic in-stent restenosis was found. Of the 3459 struts, 3315 (95.8%) were covered with neointima. The median neointimal thicknesses of the stent struts on calcified, fibrous, and lipid-rich lesions were 20 μm (interquartile range [IQR], 10–50 μm), 70 μm (40–140 μm; *P* < .001), and 90 μm (50–170 μm; *P* < .001), respectively. These differences were observed regardless of the type of second-generation DES used.

Most of the stent struts were covered with neointima. The neointimal thickness after the second-generation DES implantation had a close relationship with the preexisting atherosclerotic lesion characteristics. In this study, we found differences in arterial healing processes due to underlying plaque; therefore, evaluating the lesion characteristics by OCT may predict the risk for future restenosis and thrombosis.

## 1 Introduction

Stent implantation is a standard strategy to reduce postprocedural restenosis and thrombus occlusion rate in the current percutaneous coronary intervention (PCI) procedure. Recently, drug-eluting stents (DESs) are preferentially used rather than bare metal stents (BMSs) because they can reduce the incidence rate of long-term restenosis.^[1]^ However, stent thrombosis, a fatal complication, and long-term restenosis have been reported to occur even after DES implantation.^[2,3]^ In particular, late stent thrombosis (LST) and very late stent thrombosis (VLST) remain to be severe problems. The incidence of LST/VLST is rare (0.61%/yr).^[4]^ However, thrombosis is a serious event for most patients, with 89% of the patients sustaining myocardial infarction and a fatality rate of 42%.^[5]^

Delayed arterial healing with poor stent strut coverage has been observed using optical coherence tomography (OCT) and it is supposed to be the main risk factor for LST and VLST after DES deployment.^[6]^ Of stent struts, 18% were reported to be not covered with neointima after first-generation DES implantation.^[7]^ The ratio of uncovered stent struts was markedly reduced to 2.6% after second-generation DES deployment. Moreover, histologically, second-generation DES implantation yielded greater strut coverage with less inflammation, less fibrin deposition, and reduced the incidence rate of LST and VLST than first-generation DES implantation.^[7]^ However, complete endothelialization has not been accomplished even after using second-generation DESs.^[8]^ The neointimal thickness after first-generation DES implantation on calcified plaque-containing lesions was reported to be smaller than that on non-calcified lesions on an intravascular ultrasonography (IVUS) study.^[9]^ The neointima was also reported to grow thicker on lipid-rich lesions than on other lesions after zotarolimus-eluting stent (ZES) deployment at the 3-month follow-up OCT study.^[10]^ However, the relationship between preexisting atherosclerotic lesions, including calcified lesions, and chronic-phase neointimal thickness after second-generation DES implantation, except ZES implantation, has not been investigated using OCT.

Although the underlying processes responsible for the development of neointimal proliferation after second-generation DES implantation are likely multifactorial, we hypothesized that preexisting atherosclerotic lesions would have a close relationship with neointimal coverage after DES implantation. In the present study, we evaluated the neointimal thickness on the stent struts of lesions with different vascular intimal plaque characteristics by using OCT.

## 2 Methods

### 2.1 Study design and population

This was a Strengthening the Reporting of Observational Studies in Epidemiology (STROBE)-compliant single-center, retrospective, observational study. The study cohort comprised patients with stable angina pectoris or asymptomatic myocardial ischemia who underwent second-generation DES implantation for de novo lesion guided by OCT and routine follow-up coronary angiography and OCT between December 1, 2010, and December 31, 2015. Follow-up coronary angiography was often performed around 6 to 9 months after PCI to evaluate for stent patency. We defined stable angina as effort angina pectoris without unstable angina and asymptomatic myocardial ischemia as coronary stenosis with suspected ischemia by echocardioechography or scintigraphy. The treatment indications were determined on the basis of the guidelines for stable angina.^[1]^ Patients with acute coronary syndrome, difficulty in continuous dual antiplatelet therapy, hemodialysis (difference from the calcification in non-hemodialysis patients), and coronary lesions for which performing OCT was difficult (e.g., ostial and severe tortuous lesions) were excluded. The reason for exclusion the case of acute coronary syndrome from this examination is that the inflammatory cells and thrombus around stent struts remains for a long term, and the fully neointimal formation are delayed as a result compared with stable angina pectoris. All patients were taking 100 mg/d of aspirin and 75 mg/d of clopidogrel the day before the stenting procedures. We performed quantitative coronary angiography analysis and rate of stenosis over 75% was defined as in-stent restenosis. The study protocol was approved by our hospital's ethics committee (approval number 3780), and written informed consent was acquired from all patients.

### 2.2 OCT analysis

We performed OCT by the flush method using the Dragonfly OCT intravascular imaging system (St. Jude Medical, St Paul, MN) or FastView OFDI intravascular imaging system (Terumo, Tokyo, Japan). At OCT imaging, we used 6Fr systems and an auto-injector, a contrast medium was used OMNIPAQUE 350. The OCT pullback length included at least 1 cm over both end of stent. Heparin was used for anticoagulant. First, we used 5000 units bolus intravenous injection, and maintained activated clotting time over 250 seconds. The same type of imaging device was used during stent placement and follow-up. The thickness of the neointima on the stent strut, vascular lumen area, stent area, and thickness from the strut to the neointima surface were measured, and plaque characteristics were evaluated in the cross-section of every 1 mm interval of the stent.

On the basis of the expert-consensus OCT documents,^[11]^ we classified the plaque characteristics outside the strut into 3 components as follows: calcified, lipid-rich, and fibrous lesions. Calcified lesions were defined as circumferential signal-poor heterogeneous regions with well-delineated borders. Lipid-rich lesions were defined as signal-poor regions with atherosclerotic plaque and poorly delineated borders. Fibrous lesions were defined as fibrous plaques with high backscattering and a relatively homogeneous signal. We evaluated the plaque characteristics before and after stent deployment, and confirmed to evaluate the stent struts at the same site before stent placement. Representative photographs of the plaque characteristics outside the strut are shown in Fig. 1 . The internal or external elastic membrane was identified on occasion in fibrous plaques, and in this study, we included a normal vessel wall or intimal thickening as this component. At the time of follow-up, we identified the same site on the basis of the position of the side branch, the distance from the end of the stent, and the shape of the plaque. We evaluated the plaque characteristics at the same site of stent placement.

**Figure 1:**
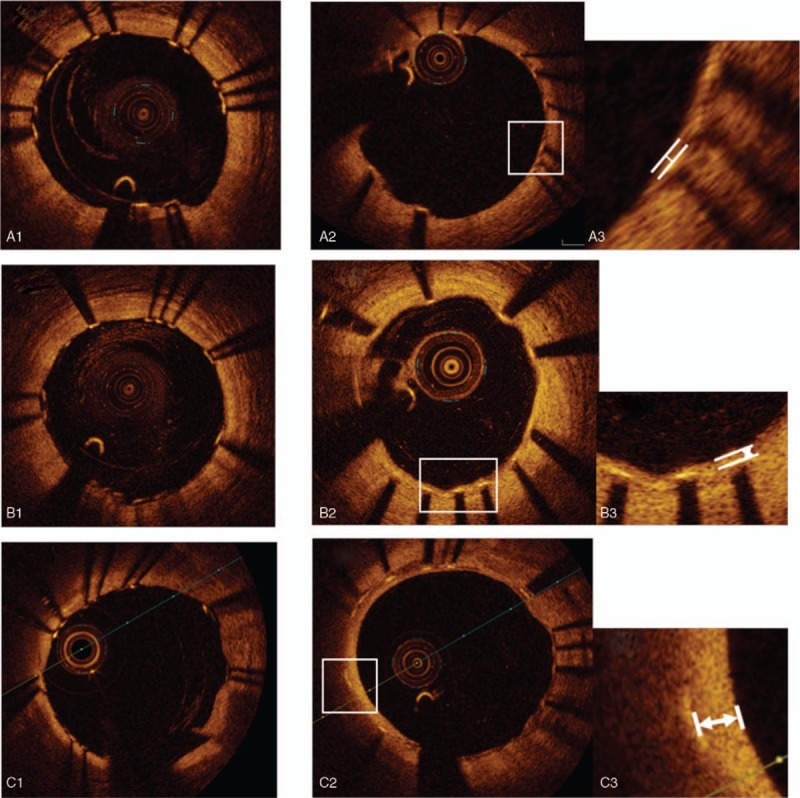
Representative optical coherence tomography (OCT) images of plaque characteristics and neointima evaluation at follow-up study. (A) Stent struts on calcified plaque. (A-1) just after stent deployment, (A-2, 3) follow up study, measured neointima thickness. (B) Stent struts on fibrous plaque. (B-1) Just after stent deployment. (B-2, 3) Follow up study, measured neointima thickness. (C) Stent struts on lipid rich plaque. (C-1) Just after stent deployment. (C-2, 3) Follow up study, measured neointima thickness. (D) Struts adjustment with plaque shape. (D-1) De novo target lesion with calcified plaque (D-2) just after stent deployment, (D-3) follow up study. (E) Struts adjustment with side branch. (E-1) De novo target lesion with side branch. (E-2) Just after stent deployment. (E-3) follow up study.

**Figure 1 (Continued):**
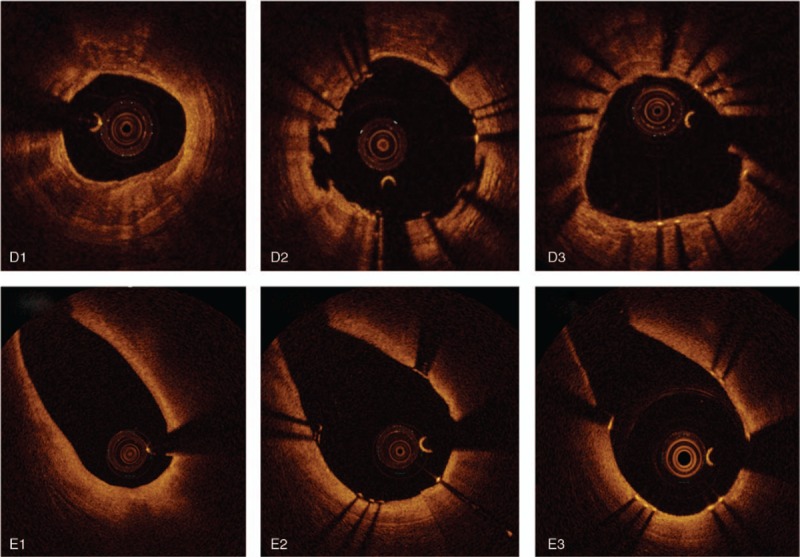
Representative optical coherence tomography (OCT) images of plaque characteristics and neointima evaluation at follow-up study. (A) Stent struts on calcified plaque. (A-1) just after stent deployment, (A-2, 3) follow up study, measured neointima thickness. (B) Stent struts on fibrous plaque. (B-1) Just after stent deployment. (B-2, 3) Follow up study, measured neointima thickness. (C) Stent struts on lipid rich plaque. (C-1) Just after stent deployment. (C-2, 3) Follow up study, measured neointima thickness. (D) Struts adjustment with plaque shape. (D-1) De novo target lesion with calcified plaque (D-2) just after stent deployment, (D-3) follow up study. (E) Struts adjustment with side branch. (E-1) De novo target lesion with side branch. (E-2) Just after stent deployment. (E-3) follow up study.

### 2.3 Statistical analysis

Statistical analysis was performed with JMP Pro 12.2.0 (SAS, Cary, NC). Continuous variables are described as mean (standard deviations) or median (interquartile range [IQR]). Nonparametric parameters were analyzed using the Wilcoxon or Kruskal-Wallis test, and are expressed as median (IQR). *P* values of <.05 were regarded as statistically significant.

## 3 Results

To investigate the relationship between preexisting atherosclerotic lesions and neointimal thickness on the stent struts, we analyzed 3459 struts from 20 stents in 15 patients. The patients’ baseline characteristics are summarized in Table 1. The coronary risk factors identified were as follows: hypertension in 86.7%, diabetes mellitus in 66.7%, dyslipidemia in 93.3%, and smoking in 53.3% of the patients. Among the patients, 86.7% and 66.7% were receiving statins and beta-blockers, respectively. The mean follow-up period after stent deployment to follow-up OCT was 264 days.

**Table 1:**
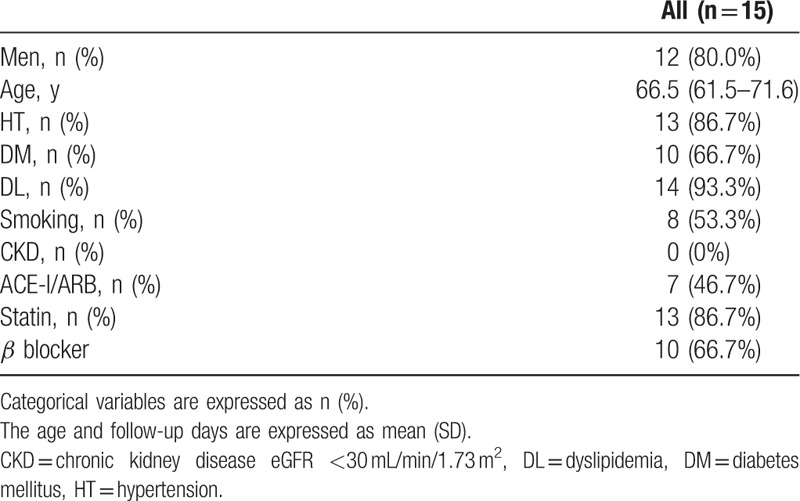
Baseline clinical characteristics.

The following stents were used: Nobori biolimus-eluting stents (BESs; Terumo; n = 7), Xience everolimus-eluting stents (EESs; cobalt-chromium [Co-Cr] EES, Abbot Vascular, Santa Clara, CA; n = 9), and Promus premier EESs (platinum-chromium [Pt-Cr] EESs; Boston Scientific, Marlborough, MA; n = 4). Vessel lesion and American Heart Association (AHA) type, median stent size and length, and the ratio of the plaque characteristics under the struts are shown in Table 2. In the OCT evaluation at the time of stent deployment, the median under-expansion ratio was 0.90, the mal-apposed strut ratio was 1.7%, and the median mal-apposed distance was 190 μm.

**Table 2:**
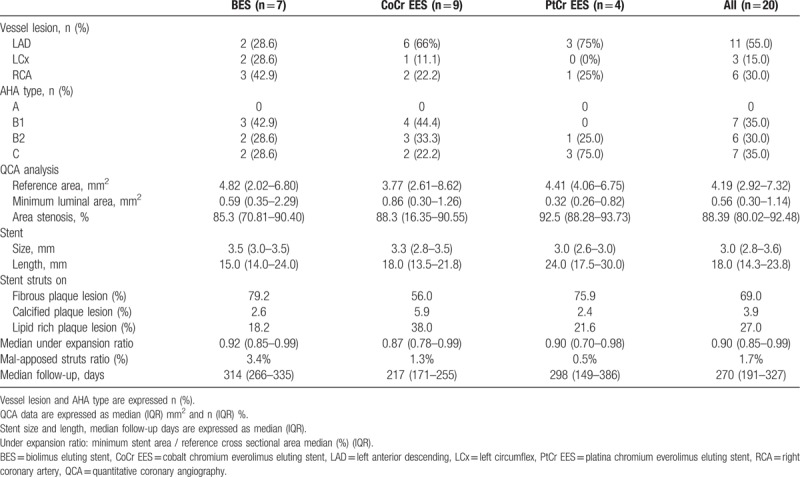
Baseline stent's characteristics.

**Figure 2:**
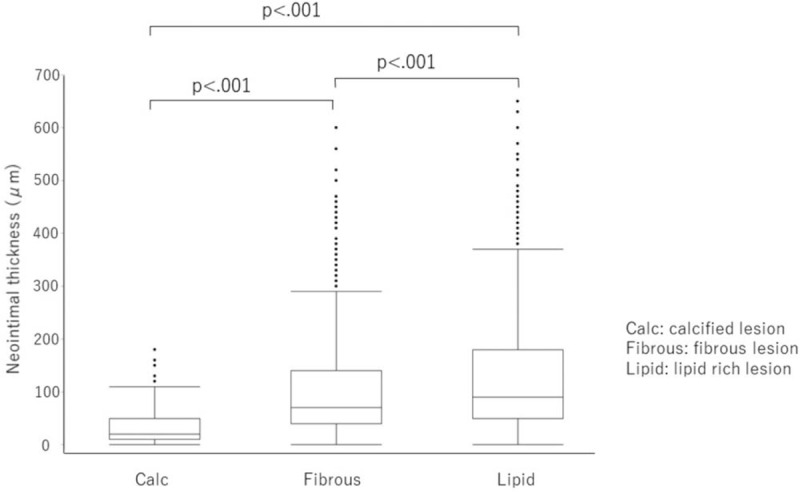
Relationship between neointimal thickness and strut's legion characteristics. The comparison of neointimal thickness of stent struts on calcified, fibrous, and lipid rich lesions. Data were expressed as median (interquartile range IQR). The box plot means median neointimal thickness (IQR). Calcified lesion: 20 μm (IQR 10–50 μm), fibrous lesion: 70 μm (IQR 40–140 μm), lipid rich lesions: 90 μm (IQR 50–170 μm).

In the present study, there were no cases of angiographic in-stent restenosis after DES implantation. OCT findings are shown in Table 3. Of the 3459 struts, 3315 (95.8%) were covered with neointima (median, 70 μm; IQR, 40–140 μm). The neointimal thickness of the struts on the non-calcified lesions (fibrous and lipid-rich lesions) was 80 μm (IQR, 40–140 μm). The neointimal thickness of the calcified plaque lesion was thinner than that of the non-calcified lesion (20 μm vs 80 μm; *P* < .001). The median neointimal thicknesses of the stent struts on calcified, fibrous, and lipid-rich lesions were 20 μm (IQR, 10–50 μm), 70 μm (IQR, 40–140 μm), and 90 μm (IQR, 50–170 μm), respectively. Statistically significant differences in median neointimal thickness on the stent struts were found among the calcified, fibrous, and lipid-rich lesions (Fig. 2). The neointimal thickness of the stent struts on the calcified lesions was thinner than that of the struts on fibrous and lipid-rich lesions (20 μm vs 70 μm, *P* < .001; 20 μm vs 90 μm, *P* < .001). The neointimal thickness of the struts on the lipid-rich lesions was thicker than that of the struts on the fibrous lesions (90 μm vs 70 μm, *P* < .001). These differences were observed regardless of the type of second-generation DES (Table 4). No significant relationship was found between neointimal thickness and follow-up period. The multivariate analysis of neointimal thickness revealed that diabetes had a weak correlation with *R* = 0.20, and statin use had a weak correlation with *R* = 0.28. In the comparison of the types of stent neointimal thickness, a weak correlation was found with Co-Cr EES and others (*R* = −0.23), and with Pt-Cr EES and others (*R* = 0.20). Stent diameter, lesion characteristics, under-expansion ratio, and malapposition were not correlated with the thickness of the neointima.

**Table 3:**
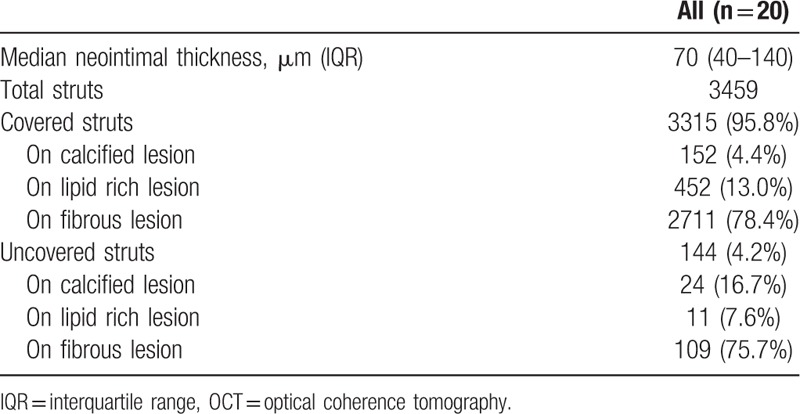
OCT findings at follow up.

**Table 4:**
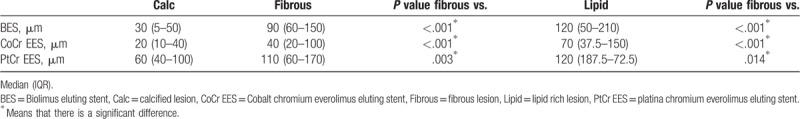
Relationship between neointimal thickness and strut's legion characteristic.

## 4 Discussion

### 4.1 Pathophysiology of neointimal formation after DES implantation

In the present study, we investigated the relationship between neointimal thickness after second-generation DES implantation and the characteristics of preexisting atherosclerotic lesions by using OCT. The main findings of this study are as follows: most of the struts were covered with neointima after second-generation DES implantation; the median neointimal thickness on stent struts was statistically significantly different among the calcified, fibrous, and lipid-rich lesions; and these differences were observed regardless of the type of second-generation DES.

Numerous studies have demonstrated that in-stent restenosis is caused by neointimal proliferation.^[12]^ The neointima is mainly composed of smooth muscle cells, and early thrombus formation and acute inflammation are followed by neointimal growth after stent implantation.^[13]^ Medial injury and lipid core penetration through struts further exacerbate inflammation.^[14]^ Our group showed the important roles of monocytes and neutrophil infiltrations in the development of exuberant neointimal proliferation of in-stent restenosis.^[13,15–18]^ Infiltrated monocytes and neutrophils, and activated platelets have been shown to promote low-density lipoprotein oxidation,^[19]^ which stimulates the proliferation and migration of smooth muscle cells via induction of the platelet-derived growth factor.^[16,18,20]^ To reduce neointimal formation, the DES releases drugs that impede smooth muscle proliferation and migration; however, these drugs also impair the normal healing process of the injured arterial wall.^[21,22]^

### 4.2 Relationship between neointimal thickness and underlying plaque of preexisting atherosclerotic lesions

As the conditions of the endothelium differ depending on the plaque characteristics, we can reasonably speculate that preexisting atherosclerotic lesion characteristics affect neointimal formation after stent placement. A previous study showed that the neointimal volume in the calcified plaque-containing cross-section was smaller than that in other lesions in an IVUS study.^[9]^ The neointima was also reported to grow thicker on lipid-rich lesions than on other lesions after second-generation DES deployment in an OCT study.^[10]^ Consistent with these reports, the present OCT study demonstrated that neointimal thickness was greater on the lipid-rich lesions than on the calcified lesions after second-generation DES implantation. Owing to the few smooth muscle cells in the calcified plaque, neointimal hyperplasia rarely occurs. On the other hand, because lipid plaque induces inflammation, more intimal proliferation is anticipated to progress. In some previous studies using angioscopy, the presence of yellow plaque was more frequently found in the implanted area of the DES than in the BMS area. Many reports also indicated that the function of regenerated endothelial cells was decreased. In addition, with scanning electron microscopy, regenerated endothelial cells after Cypher stent implantation have been reported to have a more immature shape than BMS.^[23]^ Thus, we suggest that an immature endothelial barrier allows blood lipid migration into the neointima after DES. An experimental study showed that eNOS and CD31 expression levels were lower in the injured carotid arteries than in the uninjured carotid arteries after balloon angioplasty.^[24]^ We suggest that a relationship exists between plaque characteristics before PCI and endothelial regeneration. Stent struts placed on the lesions with lipid-rich plaque are thought to have a thickened neointima. Thus, the risk for restenosis due to neointimal proliferation in the future and the possibility of the appearance of neoatherosclerosis due to neointimal proliferation may increase. Indolfi et al^[25]^ reported a case of higher neointimal formation after bioresorbable vascular scaffold (BVS) implantation. In this case, most of the neointima were well thickened, showing a heterogenous or layered pattern by OCT, although relatively thin heterogeneous neointima were partially recognized. The preexisting plaques before BVS implantation were mostly lipid-rich plaques with a necrotic core.

Coronary calcification is known to be associated with advanced age; sex; smoking; and the presence of diabetes mellitus, hypertension, and renal dysfunction.^[26,27]^ In addition, insufficient stent expansion, stent malapposition, and polymer damage of DESs sometimes occur in calcified lesions after PCI.^[28]^ These phenomena were suggested to lead to less acute lumen gain and more late lumen loss because of excessive neointimal proliferation.^[28]^ By contrast, the risk for stent thrombosis after stent deployment in calcified lesions is potentially higher than that in non-calcified lesions.^[29]^ Additional histological studies have also revealed that the most powerful predictor for stent thrombosis is insufficient, immature endothelial coverage.^[6]^ Retardation of re-endothelialization on the stent strut in calcified lesions can be the cause of the neointimal proliferation that follows DES implantation. In the present OCT study, we found that the neointimal thickness of stent struts in calcified lesions was thinner than that in non-calcified lesions after stent implantation. These results suggest retardation of the re-endothelialization on stent struts in calcified lesions.

### 4.3 Clinical implication

The ratio of the neointima coverage was increased with the second-generation DES as compared with the first-generation DES.^[7]^ However, our present findings indicate that not all struts were homogeneously neointimalized even after deploying the second-generation DES and that the thickness of the neointima was affected by the preexisting plaque characteristics. These findings suggest that the difference in characteristics of the plaque under the strut causes a heterogeneous neointimal development and increases the risk for restenosis. Moreover, insufficient neointimal development in the calcified lesion could cause thrombosis in the chronic phase. Evaluating the nature of the preexisting lesion during stent placement may predict the risk for future restenosis and thrombosis.

### 4.4 Limitations

This study had several limitations. First, because this study was retrospective and included a small number of patients, a hypothesis was proposed on the basis of the study results. In addition, the follow-up period had some variations, the clustering effects were difficult to adjust, and selection bias could not be completely excluded. Second, during the OCT analysis, the post-stent deployment and follow-up stent lesions were not completely the same. Third, although malapposed strut distance and ratio may be related to the neointimal thickness, we could not analyze the aforementioned relationship because of the small number of malapposed struts. Fourth, neointimal tissues were analyzed using OCT but were not classified using pathological methods. Thus, prospective studies that include more cases and pathological examination are needed. Finally, in our study, the proportion of calcified plaques was low, and that of lipid-rich or fibrous plaques was high. The influence of such plaque distribution cannot be completely denied statistically.

### 4.5 Conclusions

In conclusion, neointimal coverage after second-generation DES implantation has a close relationship with preexisting atherosclerotic lesion characteristics. Evaluating the lesion characteristics during stent placement using OCT may predict the risk for future restenosis and thrombosis.

## Author contributions

**Conceptualization:** Kenichi Sugioka, Makiko Ueda.

**Investigation:** Yohta Nomoto, Masashi Nakagawa, Nobuyuki Shirai.

**Project administration:** Masashi Nakagawa.

**Supervision:** Keiko Kajio, Kazuki Mizutani, Takanori Yamazaki, Kimio Kamimori, Yasuhiro Izumiya, Minoru Yoshiyama.

**Validation:** Yohta Nomoto, Masashi Nakagawa.

**Writing – original draft:** Yohta Nomoto.

**Writing – review & editing:** Yasuhiro Izumiya, Minoru Yoshiyama.

Yohta Nomoto orcid: 0000-0001-5153-9071.

Yasuhiro Izumiya orcid: 0000-0003-2332-9151.

Minoru Yoshiyama orcid: 0000-0002-3197-0494.
